# Influenza response planning for the centers of excellence for influenza research and surveillance: Science preparedness for enhancing global health security

**DOI:** 10.1111/irv.12742

**Published:** 2020-04-19

**Authors:** Kristine A. Moore, Julia T. Ostrowsky, Angela J. Mehr, Michael T. Osterholm, The CEIRS Pandemic Planning Committee, Richard W Compans, Adolfo García‐Sastre, Walter A Orenstein, Andrew Pekosz, Daniel R Perez, Richard E Rothman, Lauren M Sauer, Stacey L Schultz‐Cherry, John Steel, David J Topham, John J Treanor, Richard J Webby

**Affiliations:** ^1^ Center for Infectious Disease Research and Policy (CIDRAP) University of Minnesota Minneapolis MN USA

**Keywords:** disease outbreaks, influenza, influenza A virus, influenza vaccines, National Institute of Allergy and Infectious Diseases, National Institutes of Health, pandemics, public health practice, public health preparedness, science preparedness

## Abstract

**Background:**

The Centers of Excellence for Influenza Research and Surveillance (CEIRS) network, funded by the US National Institutes of Health, has been operational since 2007 and is tasked with conducting research to improve understanding of influenza viruses. Recently, CEIRS developed an Influenza Response Plan (IRP) to improve science preparedness for the network.

**Methods:**

Development of the IRP involved a collaborative process between project staff, CEIRS center directors or their designees, and NIAID CEIRS leadership (referred to as the Pandemic Planning Advisory Committee [PPAC]). Project staff identified and summarized the response capabilities of each center and then worked with the PPAC to identify and rank research priorities for an emergency response using a modified Delphi method.

**Results:**

Key elements of the response plan include tables of response capabilities for each CEIRS center, a framework that outlines and ranks research priorities for CEIRS during an emergency situation, and an operational strategy for executing the research priorities.

**Conclusions:**

The CEIRS IRP highlights the importance of enhancing science preparedness in advance of an influenza pandemic or other influenza‐related zoonotic incident to ensure that research can be carried out expeditiously and effectively in emergency situations and to improve global health security.

## 1 BACKGROUND

Over the past 20 years, the world has witnessed a number of global or regional infectious disease emergencies, including the outbreak of Severe Acute Respiratory Syndrome coronavirus (SARS‐CoV) in 2002‐2003, the H1N1 influenza pandemic in 2009‐2010, the emergence of Middle Eastern Respiratory Syndrome coronavirus (MERS‐CoV) in 2012, the Zika virus epidemic in the Americas in 2015‐2016, the West Africa Ebola virus epidemic in 2015‐2017, and most recently, the 2019‐2020 international outbreak of coronavirus disease 2019 (COVID‐19; caused by SARS‐CoV‐2).^1^, ^2^, ^3^, ^4^, ^5^, ^6^, ^7^ While these situations have required broad‐scale public health emergency responses, they also illustrate the need for coordinated, rapid‐response scientific research and stronger processes to promote science preparedness in advance of infectious disease emergencies. According to the US Office of the Assistant Secretary for Preparedness and Response (ASPR) within the US Department of Health and Human Services (HHS), science preparedness “is a collaborative effort to establish and sustain a scientific research framework that can enable emergency planners, responders, and the whole community to better prepare for, respond to, and recover from major public health emergencies and disasters.”^8^ Furthermore, in a public health emergency, scientists may need to “operate within a finite window of opportunity to identify, collect, and analyze critical and time‐sensitive data that may be available only during the immediacy of the event or incident,” which highlights the importance of preparedness efforts.^8^ Recent publications on science preparedness have focused on the need to develop plans (eg, protocols and approvals from Institutional Review Boards) for initiating clinical research during public health emergencies, particularly regarding the inclusion of pregnant women and children in clinical trials of candidate medical countermeasures during outbreaks.^9^, ^10^ A need also exists, however, to ensure rapid initiation of basic science research during outbreaks of novel or emerging pathogens, particularly with regard to developing new medical countermeasures.

Because of the constant and unpredictable threat of an influenza pandemic, preparedness to conduct research during situations involving pandemic influenza or emergence of novel influenza viruses with pandemic potential remains an important priority for enhancing overall science preparedness and global health security. While not caused by a novel influenza virus, the current COVID‐19 outbreak has many parallels to pandemic influenza and clearly demonstrates the importance of rapid mobilization of critical scientific research as part of the overall response. To strengthen capabilities for scientific research on influenza, in 2007 the US National Institutes of Health (NIH) established the Centers of Excellence for Influenza Research and Surveillance (CEIRS) network. Since the program was initiated, NIH has funded six multidisciplinary research centers across the United States, with a number of associated international partners. Currently, five centers are funded through CEIRS.^11^ By conducting animal and human influenza surveillance and influenza pathogenesis and host response research, the CEIRS network is expected to fill key knowledge gaps, enabling accelerated development of new and improved control measures against influenza. In addition, the CEIRS centers are expected to serve as resources for the scientific community and to provide a research response (including production of essential reagents) in the event of an emergency influenza situation. Finally, the CEIRS network also has critical research capabilities that can be leveraged for responding to outbreaks involving other novel respiratory pathogens, which was done with MERS in 2012 and is now ongoing with COVID‐19.

The CEIRS network has been highly successful in advancing the field of influenza research, as evidenced by the extensive number of publications produced by CEIRS investigators over the past 13 years (more than 1700 publications between 2007 and early 2018).^11^ In addition, the CEIRS network has demonstrated its ability to rapidly redirect research in response to emergency situations, as shown during the 2009‐2010 H1N1 pandemic, which is a strong testament to the value of having a coordinated research infrastructure and flexible funding mechanism in place before the onset of a public health emergency. While the CEIRS network made significant scientific contributions during the 2009‐2010 pandemic, several organizational improvements were later identified that could enhance the CEIRS response in future. One key issue was the need to have a mechanism in place to provide an up‐to‐date assessment of the technical response capabilities of each CEIRS center, which could serve as an easy reference during emergency situations. A second issue was the need for a more standardized approach to coordinating the research of CEIRS investigators during emergencies, using a pre‐defined research prioritization scheme. A third was the need to more clearly define the role of the NIAID CEIRS project officer (also referred to as the director of operations) during an emergency response. To address these concerns, the CEIRS centers worked collaboratively to develop the CEIRS Influenza Response Plan (IRP). This report briefly outlines the steps in that process and highlights the unique elements of the CEIRS IRP that focus on successfully executing the scientific mission of the CEIRS network during an emergency situation.

## 2 METHODS

The first step in this project was to create the CEIRS Pandemic Planning Advisory Committee (PPAC), which included center directors and/or at least one senior researcher from each center, NIAID CEIRS leadership personnel, and project staff (contracted from the Center for Infectious Disease Policy and Research [CIDRAP] at the University of Minnesota). The committee's charge was to provide critical feedback and guidance regarding the process and outcome of CEIRS pandemic research planning, including review of interim and final products. During the course of the planning process, the PPAC met regularly to discuss various aspects of CEIRS influenza response planning and to provide critical input on overarching planning issues.

The second step involved collecting background information from each CEIRS center on its research capabilities and areas of expertise relevant to rapid initiation of pandemic research and response. Project staff reviewed the work of each center, including publications, project plans, and other available written information, and identified overarching themes for capabilities in two areas. The first—capability to provide technical resources—included the following themes: enhancing situational awareness of novel viruses, reagent production and distribution, ability to perform high‐quality analytics, availability of biocontainment facilities, ability to perform human cell culture, public communication, availability of clinical protocols, tools for information sharing, and other capabilities not identified elsewhere. The second—rapid initiation of pandemic research—included the following themes: epidemiologic analysis and surveillance research (as appropriate for CEIRS and not in conflict with other US government agencies, such as the Centers for Disease Control and Prevention [CDC]), environment/ecology assessment, virus characterization, analysis of host factors, vaccine development, influenza therapeutics, development of new methodologies and protocols, and other relevant resources or expertise. Specific capabilities for each theme were then determined, based on a review of information provided by the centers. These themes and capabilities were summarized in two table formats, and each center was asked by project staff to complete the tables with information specific to their center. Project staff then reviewed the information and combined it into two large tables to reflect capabilities for all centers. Each center director verified the information to ensure its accuracy. These tables were initially compiled in 2017 and have been updated annually since; they provide an easy up‐to‐date mechanism to quickly identify center capabilities.

The next step involved developing a framework for prioritizing research activities during future pandemic incidents or situations involving the emergence of novel influenza viruses with pandemic potential. This framework identifies the key research questions for CEIRS to address during a pandemic or pre‐pandemic situation and prioritizes the associated research activities. Project staff developed a draft framework of research questions and activities and convened an in‐person meeting of the PPAC to review and prioritize them. The prioritization process involved a modified Delphi method, whereby PPAC members were asked to individually rank activities as high or medium priority and as readily feasible (ie, the activity can be completed in a timely fashion and will not require additional resources) or not readily feasible (ie, the activity will take longer, will require additional resources, or may not be feasible). The prioritization rankings were then added to the framework to reflect the input of PPAC members. A revised version of the framework was resubmitted to PPAC members for a second review and then was discussed in detail during another in‐person PPAC meeting to develop consensus among the group.

## 3 RESULTS: KEY ELEMENTS OF THE CEIRS IRP

The CEIRS IRP includes several elements unique to the scientific mission of CEIRS; the full version of the plan is available on the CEIRS website.^12^ First, the CEIRS IRP provides detailed information on the research capabilities of each of the CEIRS centers with regard to the ability to rapidly provide technical resources and the expertise necessary to quickly initiate new influenza research studies. This information is summarized in the tables described above, so that response coordinators can easily assess and review the capabilities of each center.

Second, the plan includes a response framework (Table 1) aligned with the broad activities that the US government assigns to the NIH during an influenza pandemic, as outlined in the original 2005 US HHS Pandemic Influenza Plan.^13^ While this framework provides a useful point of reference, the actual research priorities will need to be determined in real time, based on a number of factors related to the situational assessment, such as how much information is available regarding the genomic and functional characteristics of the virus and the epidemiologic features of the associated infection (eg, global distribution, disease occurrence in human and animals, and disease severity).

**Table 1:** CEIRS network influenza response research framework

NIH roles and responsibilities for pandemic response^a^	Research questions applicable to the CEIRS network for an influenza response	Primary research activities for the CEIRS network that apply to the research questions
Goal 1: Provide technical and infrastructure resources to support USG and global efforts from international organizations to respond to the pandemic^b^		
Prepare reference strains appropriate for vaccine manufacturing.	[Not applicable.]	Generate, amplify, and disseminate prototype virus stocks, including stocks of vaccine candidate viruses. (HP, RF) Generate panels of relevant virus clones. (MP, RF) Generate mouse‐adapted viral strains. (MP, RF) Clone gene segments into reverse genetics vectors and protein expression plasmids. (MP, RF) Create other key reagents (eg, monoclonal antibodies, assays, antisera, viral antigens, recombinant plasmids, proteins, and peptides). (HP, RF)
Goal 2: Provide scientific information to support the rapid development and use of medical countermeasures (eg, diagnostics, therapeutics, and vaccines) to mitigate the impact of an influenza pandemic^a^		
Develop improved drugs against influenza.	Are investigational drugs safe and effective against the pandemic virus? Is the pandemic virus sensitive to existing antiviral agents? If the pandemic virus is sensitive to existing agents, is it likely to develop resistance to antiviral agents over time?	Conduct pre‐clinical evaluation of investigational drugs in animal models. (MP, RF) Determine antiviral susceptibility of pandemic strains to FDA‐approved and investigational drugs, using sequence and functional analyses. (HP, RF) Assess acquisition of antiviral resistance for pandemic strains over time. (MP, RF)
Evaluate the immune response to infection and vaccination	What is the innate immune response to the pandemic virus in animals and humans? What are the cell‐mediated and humoral immune responses to the pandemic virus in animals and humans? What is the baseline level of pre‐existing immunity to the pandemic virus in humans in different geographic areas and in different age groups? Do current influenza vaccines (including stockpiled vaccines) protect against infection with the pandemic virus? How does the immune response generated by the current seasonal vaccine relate to the pandemic strain? What are the immunologic responses to candidate vaccines in animals and humans?	Analyze innate and adaptive immune responses to the pandemic virus in animal models and in humans. (MP, RF) Assess baseline levels of pre‐existing immunity to the pandemic virus in humans using different markers. (HP, RF) Determine efficacy of current vaccines (including stockpiled vaccines) against the pandemic virus. (HP, NRF) Identify cross‐reactive antibodies and assess cross‐reactive immune responses to the pandemic virus. (MP, NRF) Determine the immunologic responses to candidate vaccines in animals and humans. (HP, RF [although somewhat time‐consuming])
Develop and clinically evaluate novel influenza vaccines and vaccination strategies (eg, adjuvants and delivery systems)	What are the growth and cytopathic effects of the pandemic virus in various cell lines and in eggs (for vaccine production)? What are the efficacies of candidate vaccines in pre‐clinical models? What are the safety, efficacy, and immunogenicity parameters of candidate vaccines in different human populations? What is the effect of prior exposure to seasonal viruses and seasonal vaccines on the efficacy of a pandemic vaccine?	Determine and improve the growth and cytopathic characteristics of the pandemic virus in cell lines and eggs. (HP, RF) Evaluate vaccine candidates. (HP, RF) Assess efficacy of candidate vaccines in pre‐clinical animal models. (HP, NRF) Assess immunogenicity, efficacy, and safety of candidate vaccines in different human populations. (MP, NRF) Determine the effect of prior exposure to seasonal viruses and seasonal vaccines on efficacy of pandemic candidate vaccines. (MP, NRF)
Develop sensitive, specific, and rapid diagnostic tests for influenza	What point‐of‐care diagnostic tests are the most sensitive and specific for rapid diagnosis of pandemic influenza?	Evaluate point‐of‐care diagnostic tests to enhance the use of antivirals. (MP, RF) Collect clinical specimens for future evaluation of diagnostic tests. (MP, RF)
Goal 3: Provide scientific information to aid in understanding the pandemic virus and assessing the potential severity of an influenza pandemic caused by the new virus^a^		
Determine the molecular basis of virulence in humans and animals	What are the determinants of virulence for the pandemic virus? What is the pathogenicity of the pandemic virus in humans and animals? Does the pandemic virus have distinct phenotypic variants that are associated with virulence or pathogenicity? Are there virologic or host factors that predict disease severity? What are the changes in the pandemic virus over time, and do these changes affect disease severity?	Assess molecular signatures/markers that influence virulence of the pandemic virus. (HP, RF) Determine pathogenicity of the pandemic virus in animal species (eg, wild birds, poultry, ferrets, mice, macaques, swine, and guinea pigs). (HP, RF) Determine phenotype in primary cells and cell lines (eg, NHBE, HTBE, and MDCK cells). (HP, RF) Assess receptor binding specificity, cleavage, and membrane fusion properties of the pandemic virus. (HP, RF) Determine neuraminidase and polymerase activity of the pandemic virus. (HP, RF) Perform phenotypic characterization of the pandemic virus in various animal models. (HP, RF) Look for virologic and host factors that are markers or predictors of disease severity. (HP, NRF) Assess impact on virulence from mutations, reassortment with other avian influenza viruses, or changes in host tropism. (HP, NRF)
Support basic research, including structure/function studies of influenza virus proteins with the goal of identifying new therapeutic targets	What are the genetic characteristics of the pandemic virus? What are the antigenic characteristics of the pandemic virus?	Perform rapid genomic sequencing and full‐genotypic characterization of the pandemic virus. (HP, RF) Perform genotyping and phenotyping of variant viruses. (MP, RF) Perform antigenic cartography of pandemic strains. (MP, RF)
Goal 4: Provide scientific information to aid in understanding and limiting transmission of the pandemic virus^a^		
Evaluate the molecular and/or environmental factors that influence the transmission of influenza viruses, including drug‐resistant strains	How transmissible is the pandemic virus in animal models? How well does the virus transmit to different animal hosts? How transmissible is the pandemic virus in humans? What are the features of the virus that make it transmissible between humans? What are the host determinants of transmission? What are the primary routes of transmission? What is the transmissibility of pandemic strains with drug‐resistant mutations? How effective would various intervention strategies be in controlling transmission?	Determine infectivity/transmissibility of the pandemic virus in different animal models, taking into consideration the limitations of extrapolating animal data to humans. (HP, RF) Determine transmission phenotypes of the virus and virus variants in animal models. (HP, RF) Determine viral tropism and replication in animal cell lines. (MP, RF) Determine viral tropism and replication in human nasal, tracheal, and lung epithelial cells. (MP, RF) Determine host factors involved in viral replication and release. (MP, NRF) Conduct epidemiologic studies to determine transmission dynamics in humans. (HP, NRF) Conduct modeling to determine transmission patterns in humans. (MP, RF) Determine transmissibility of antiviral‐resistant strains. (MP, NRF) Use computational modeling studies to predict the potential effectiveness of alternative control strategies (eg, school closures, other social distancing measures, and travel restrictions). (HP, NRF)
Study the evolution and emergence of influenza viruses, including the identification of factors that affect influenza host range and virulence	What is the likely origin (species, geographic location) of the virus? What is the relatedness of the pandemic virus to seasonal influenza viruses? What is the potential for reassortment of the pandemic virus with seasonal viruses? What are the rates of infection in various animal species? What are the rates of infection in various human populations in different geographic areas? Which human populations are at highest risk of increased morbidity and mortality? What are the predictions for global or regional disease spread based on epidemic models and simulations? What are the predictions for disease spread among high‐risk populations and healthcare providers based on epidemic models? Does the acquisition of antiviral resistance affect viral fitness?	Conduct phylogenetic analysis of the pandemic virus to elucidate virus origin and evolution. (MP, RF) Determine relatedness of the pandemic virus to current seasonal vaccine viruses. (HP, RF) Determine potential for reassortment of the pandemic virus with contemporary human seasonal viruses. (MP, NRF) Assess virus adaptation in various hosts. (MP, NRF) Conduct epidemiologic studies to determine disease burden in different populations, geographic spread, and disease severity. (HP, RF) Conduct computational modeling of the pandemic virus to determine emergence and spread over time and predict impact. (MP, RF)
Support virologic and serologic surveillance studies in animals of the distribution of influenza viruses with pandemic potential	What are the rates of infection in animal species of interest? What are the rates of infection in humans at the human‐animal interface? What is the viral ecology and environmental stability of the pandemic virus?	Conduct surveillance studies in animal populations of interest (eg, wild birds, poultry, and swine [domestic and wild]). (MP, RF) Conduct surveillance of animal‐to‐human and human‐to‐animal transmission. (HP, RF) Conduct environmental and ecological assessments of the pandemic virus. (MP, RF) Assess environmental stability of pandemic strains. (MP, RF)

Definitions:

HP: Highest priority; MP: medium priority.

*HP:* Provides critical scientific information or a critical service for the USG response to either an influenza pandemic or a pre‐pandemic situation and the information/service is urgently needed.

*MP:* Provides critical scientific information that will be useful for pandemic/pre‐pandemic response but the information is not urgently needed.

RF: Readily feasible; NRF: Not readily feasible or feasibility may not be clear.

*RF:* The activity can be completed in a timely fashion and will not require additional resources.

*NRF:* The activity will take longer, will require additional resources, or may not be feasible.

Abbreviations: FDA, US Food and Drug Administration; HTBE, human tracheobronchial epithelial cells; MDCK, Madin‐Darby canine kidney epithelial cells; NHBE, normal human bronchial epithelial cells.

^a^ According to the HHS Pandemic Influenza Plan (HHS 2005), the NIH directs these broad activities during an influenza pandemic. Some of these activities are central to the mission of the CEIRS network, while other activities are more peripheral to CEIRS, and CEIRS investigators may have a less substantial role (or a smaller supporting role) than other entities.

^b^ These goals are specifically intended for response to an influenza pandemic, but are also applicable to emergence of a novel influenza strain with pandemic potential.

Third, the plan provides a concept of operations for the CEIRS network to (1) rapidly generate scientific and clinical information about a pandemic or novel influenza virus with pandemic potential, (2) provide technical resources to support NIAID’s response both nationally and internationally, (3) coordinate effectively to promote information sharing and situational awareness, and (4) enhance efficiency of research during a pandemic or pre‐pandemic incident. This concept of operations is notionally based on the primary functions for emergency response as outlined in a traditional Incident Command System (ICS) model^14^; however, in this situation, the model is intended to accommodate a scientific research mission as opposed to a more traditional public safety mission. In this response model, the major “strategy” for response involves identification of the situationally dependent research priorities for the CEIRS network. The “tactics” for response are the individual scientific projects that need to be completed by the CEIRS centers to accomplish the key elements of the “strategy” (ie, achieve the research priorities and fulfill the scientific mission).

The CEIRS IRP concept of operations identifies the NIAID CEIRS director of operations as the person responsible for coordinating the CEIRS response, within the context of the broader NIAID response and under the direction of senior NIAID leadership. The CEIRS IRP model incorporates the additional ICS functions of planning, operations, logistics, and finance/administration, but places them within the limited context of CEIRS. These functions will be provided by NIAID staff, and those personnel, along with the CEIRS director of operations, will collectively constitute the CEIRS Influenza Response Leadership Team. The model places the CEIRS researchers under the “operations” function, as the primary “responders” for completing the research mission.

The CEIRS IRP concept of operations also includes a Pandemic Response Advisory Committee (PRAC) comprised of the CEIRS center directors or the designees. The purpose of the PRAC is to assist and advise the CEIRS director of operations and the CEIRS leadership team in identifying the research priorities applicable to the specific situation, given their extensive research expertise, using the research framework provided in the CEIRS IRP as a point of reference. The PRAC also will be able to help identify barriers and challenges in implementing the response, develop strategies for addressing such challenges, serve as an avenue of communication back to the centers, and serve as a forum for resolving any issues or conflicts that arise among the CEIRS centers during the incident response. The plan indicates that the PRAC will meet with CEIRS Influenza Response Leadership Team regularly throughout the course of the incident.

Once research priorities appropriate to the situation have been established by NIAID CEIRS leadership, with technical input from the PRAC, they will be communicated to the CEIRS centers. Each center will then be asked to provide a specific research plan that aligns with the defined research priorities that have been approved by NIAID leadership. These research plans will be reviewed by NIAID to ensure that: the plans are consistent with the role of CEIRS in a research response, they align with the research priorities specific to the incident, and the level of redundancy across centers is appropriate. NIAID will determine which plans or aspects of the plans will be funded (through existing or new contracts) in order to fulfill NIAID’s expectations for the role of the CEIRS centers during the particular emergency situation. This approach offers an efficient mechanism to rapidly identify and move forward with a carefully determined set of critical research priorities. It also allows the CEIRS centers to provide their expertise in defining the priorities, but leaves the ultimate decision‐making to NIAID leadership, in accordance with the contractual agreements between the US government and the CEIRS centers.

## 4 DISCUSSION

According to ASPR, science preparedness involves five key elements: coordination and integration, scientific research, research infrastructure, public health practice, and emergency management.^8^ Several of these elements apply to the CEIRS role in responding to a public health emergency involving a novel or emerging influenza virus (or potentially other respiratory virus of concern, such as MERS‐CoV or SARS‐CoV‐2), including coordination and integration, scientific research, and research infrastructure. The CEIRS network serves as a flexible infrastructure that is able to accommodate the research needs that are critical components of the response; this infrastructure provides a mechanism for funding, ensures availability of research scientists, and establishes organizational capacities to implement key research as needed. The CEIRS IRP adds an additional level of refinement to the CEIRS response by identifying the scientific capabilities of each CEIRS center in an easy‐to‐access reference tool, (2) providing a framework of pre‐defined research priorities that can be tailored to the unique features of the situation, and (3) providing an operational model to promote efficiency. It is important to note that use of ICS principles in the CEIRS IRP is notional and does not imply that CEIRS will “fully activate” an official ICS structure. Rather, the CEIRS IRP is consistent with the defined roles of CEIRS and does not conflict with other existing lines of authority within HHS or other elements of Emergency Support Function 8 (ESF 8) in the US Emergency Response Framework.^15^

While the CEIRS IRP is an important step in promoting science preparedness for the CEIRS network, it does not address a number of other key needs, such as having clinical trial protocols in place in advance of an emergency, rapid access to viral samples, generation of interagency agreements, or challenges with international memoranda of understanding (MOUs) and approvals. These issues were determined to be outside the scope of the current plan, but are essential to optimal scientific preparedness for the CEIRS network. CEIRS leadership (NIAID personnel and the CEIRS center directors or their designees) need to continue to identify remaining gaps and specific steps to address them, and create an overall strategy to ensure that they are addressed to the degree feasible.

The 2017 update to the HHS Pandemic Influenza Plan identifies science infrastructure and preparedness as one of the seven key domains for improving pandemic response capacity between 2017 and 2027.^16^ The update specifically mentions the valuable role of the CEIRS network in influenza research. According to the update, “HHS’s fundamental investments in science infrastructure and preparedness have delivered critical knowledge to understand both seasonal influenza viruses and those with pandemic potential. Establishing and sustaining scientific research frameworks, addressing the full spectrum from basic discovery through patient intervention, will dramatically improve comprehensive pandemic outbreak preparedness, response, and recovery.”

From 2017 to 2027, HHS intends to enhance science preparedness by achieving the following three goals.^16^ The first is to support scientific infrastructure preparedness, including ensuring the capacity to conduct clinical, behavioral, and epidemiologic research. This also includes issues such as safety oversight and steps to simplify collaboration, ethical practices, and evaluation activities. The second is to support basic and translational research to improve medical countermeasures and strategies to prevent, diagnose, treat, and respond to pandemic influenza. The third is to establish and sustain a scientific preparedness framework that can align and integrate public health practice and scientific research during an influenza pandemic. Research conducted by the CEIRS network is applicable to all three of these science preparedness goals, and the CEIRS IRP is an important step in ensuring that research is prioritized quickly and an overarching research approach is established early in any pandemic or potential pre‐pandemic situation, with appropriate oversight and coordination of activities by NIAID leadership.

## AUTHOR CONTRIBUTIONS

KM: Conceptualization‐Lead, Methodology‐Equal, Writing‐original draft‐Lead, Writing‐review & editing‐Lead. JO: Conceptualization‐Supporting, Methodology‐Equal, Writing‐original draft‐Supporting, Writing‐review & editing‐Supporting. AM, MO, RC, AG‐S, WO, AP, DP, RR, LS, SS‐C, JS, DJT, JT, RW: Conceptualization‐Supporting, Writing‐review & editing‐Supporting.

## APPENDIX 1

Members of the CEIRS Pandemic Planning Advisory Committee: Richard W. Compans, PhD, Emory‐University of Georgia (UGA) Center of Excellence for Influenza Research and Surveillance (Emory‐UGA CEIRS), Emory University; Atlanta, Georgia, USA. Adolfo García‐Sastre, PhD, Center for Research on Influenza Pathogenesis (CRIP), Icahn School of Medicine at Mount Sinai; New York City, New York, USA. Walter A. Orenstein, MD, Emory‐UGA CEIRS. Andrew Pekosz, PhD, Johns Hopkins Center of Excellence for Influenza Research and Surveillance (Johns Hopkins CEIRS), Johns Hopkins University; Baltimore, Maryland, USA. Daniel R. Perez, PhD, University of Georgia; Athens, Georgia, USA. Richard E. Rothman, MD, PhD, Johns Hopkins CEIRS. Lauren M. Sauer, MSc, Johns Hopkins CEIRS. Stacey L. Schultz‐Cherry, PhD, SJCEIRS. John Steel, PhD*, Emory‐UGA CEIRS (*currently with the Centers for Disease Control and Prevention, Atlanta, GA, USA). David J. Topham, PhD, New York Influenza Center of Excellence (NYICE), University of Rochester Medical Center; Rochester, New York, USA. John J. Treanor, MD*, NYICE (*currently retired). Richard J. Webby, PhD, SJCEIRS. Dr. García‐Sastre is an inventor of patents on influenza virus vaccines owned by the Icahn School of Medicine at Mount Sinai.
